# Toxicities Caused by Head and Neck Cancer Treatments and Their Influence on the Development of Malnutrition: Review of the Literature

**DOI:** 10.3390/ejihpe10040066

**Published:** 2020-10-02

**Authors:** Maddison Hunter, Jane Kellett, Kellie Toohey, Nathan M. D’Cunha, Stephen Isbel, Nenad Naumovski

**Affiliations:** 1 Faculty of Health, University of Canberra, Bruce, 2617 ACT, Australia; maddy.hunter@canberra.edu.au (M.H.); jane.kellett@canberra.edu.au (J.K.); kellie.toohey@canberra.edu.au (K.T.); stephen.isbel@canberra.edu.au (S.I.); 2 Prehabilitation, Activity, Cancer, Exercise and Survivorship (PACES) Research Group, University of Canberra, Bruce, 2617 ACT, Australia; 3 Functional Foods and Nutrition Research (FFNR) Laboratory, University of Canberra, Bruce, 2617 ACT, Australia; 4 Department of Nutrition and Dietetics, School of Health Sciences and Education, Harokopio University, 17676 Athens, Greece

**Keywords:** head and neck cancer, malnutrition, treatment, toxicities

## Abstract

Malnutrition poses a significant problem for oncology patients, resulting in fatalities within this population. Patients with head and neck cancer (HNC) are at high risk, with up to 90% developing malnutrition. Common treatments used for HNC can often lead to adverse side effects, including oral health conditions, gastrointestinal upsets, and several metabolic changes. Consequently, treatments can cause inadequate nutritional intake, resulting in a reduction in energy consumption, and alterations in energy utilization, contributing to the development of malnutrition. Furthermore, the presence of these treatment toxicities, and the related malnutrition can lead to reduced quality of life, weight loss, and psychological distress. There are interventions available (nutritional, medicinal, and physical therapies) that have demonstrated potential effectiveness in reducing the severity of symptomatic toxicities, reducing the risk of malnutrition, and improving survival outcomes of patients with HNC. Based on the findings of this review, there is an urgent need for the implementation or continuation of multi-disciplinary strategies, as well as updated and improved guidelines to assist in the prevention and treatment of malnutrition caused by treatment-related toxicities in patients with HNC.

## 1. Introduction

Cancers of the head and neck region represent a significant global health burden, with approximately 1.5 million new cases diagnosed every year, resulting in over one million deaths [1]. The term head and neck cancer (HNC) encompasses a range of neoplasms that originate in this anatomical region, including tumors of the oral cavity, upper aerodigestive tract (including the pharynx, larynx and esophageal opening), the sinuses, salivary glands, bone and soft tissue of the head and neck [2]. The incidence of HNC is increasing in developed countries, with risk factors including the use of tobacco products, consumption of alcohol, genetics, age, and viral infections such as human papillomavirus (HPV) [2,3].

The combination of prescribed treatments, their associated toxicities, and the location of the tumor place patients with HNC at high risk of malnutrition. The most common therapies used in the treatment of HNC include radiotherapy (RT), which is prescribed to target cancer cells located in this region, with chemotherapy (CT) also targeting circulating cancer cells. Although beneficial, there are several negative effects associated with RT and CT, including multiple side effects and toxicities resulting in a range of oral health complications, such as oral mucositis and xerostomia, and gastrointestinal problems [4]. Furthermore, the high incidence of these adverse reactions in this population have the potential to contribute to reduced nutritional intake and increase the risk of developing malnutrition. Patients with HNC may experience difficulty consuming food due to the physical presence of their tumors in addition to these symptoms, placing them at nutritional risk well before experiencing the side effects of active treatment [5].

While HNC represents less than 5% of cancer deaths [6], this population group is at high-risk of developing malnutrition (up to 90%) during active treatment [7]. Malnutrition is commonly experienced during oncology treatments, and is defined as “an acute, subacute or chronic state of nutrition, in which a combination of varying degrees of overnutrition or undernutrition with or without inflammatory activity have led to a change in body composition and diminished function” [8]. The diagnostic criteria of malnutrition includes the factors of; involuntary weight loss, low body mass index (BMI), reduced muscle mass, reduced food intake, and disease burden and inflammation [9]. This population experiences malnutrition due to their tumor and/or treatments directly impacting on the ability to masticate, swallow, and tolerate food [10]. Furthermore, health-related consequences of malnutrition in oncology patients can have detrimental effects on their cancer treatment outcomes, including impaired treatment response (i.e., due to reduced absorption and utilization of the medication) that can result in extended periods undergoing treatment [11]. Malnutrition can also reduce recovery following RT and CT, and contribute to an overall reduction in physical strength and quality of life (QoL) [11], with increased levels of mortality rates observed (10–20%) that are specifically related to the symptoms of malnutrition rather than the diagnosis itself [7,12].

This review aimed to identify the treatment-related causes that contribute to the development of malnutrition in patients with HNC. Malnutrition in patients with HNC is one of the fundamental risk factors associated with a reduction in QoL, prognosis and recovery during treatment. The treatment effects most likely to impact the development of malnutrition in patients with HNC are identified and discussed, particularly those responsible for a decreased consumption of a nutritionally appropriate diet. In addition, findings of this review highlight currently utilized interventions which have been demonstrated to reduce the severity of malnutrition in HNC by minimizing the treatment-related toxicities which cause its development.

## 2. Methods

Throughout 2019 and 2020, a review was completed using the Google Scholar and PubMed electronic databases to identify relevant articles. All articles selected for inclusion in this review were peer-reviewed studies in humans, and published in English after the year 2000 to incorporate the most relevant literature. Search terms were categorized based on the relevant section of the review they were to be included in. Additionally, articles were required to use the term “head and neck cancer”, with the terms “malnutrition” and “toxicity” in order to be included. Furthermore, due to the various sections and many aspects of this review, the requirements of the included articles were very broad.

In addition to discussing patients with HNC, articles were included that defined the oncology treatment received, and described the treatment-related toxicities experienced in this population. Furthermore, articles were included that described the effects of the toxicities on the development of malnutrition, and its consequential impacts.

## 3. Current Treatments for Head and Neck Cancer

The treatments used for HNC can often contribute to the development of malnutrition in patients, with the exact prescription of treatments dependent on a variety of factors (i.e., location, stage, and severity of the tumor). Treatments can include surgery, RT and CT used in isolation, or as concurrent chemo-radiotherapy (CRT) [13], with less common treatments including immunotherapy, hormonal therapy and targeted therapy [14]. These treatments also have potential disadvantages and associated toxicities, which can contribute to the development of malnutrition in patients with HNC. It should also be highlighted that patients are at risk of the development of malnutrition-related symptoms prior to the commencement of their oncology treatment, such as a reduction in weight and caloric intake, due to factors including tumor size, with treatments having the potential to exacerbate these effects [15].

### 3.1. Surgery

Surgical procedures for patients with HNC are initially utilized for primary tumors, and can be performed for preventative, curative, palliative, and reconstructive purposes, with outcomes largely dependent on the stage of the tumor at the time of surgery [16]. These can also be invasive and may result in changes to the functional properties of affected areas, such as impacts on the cranial nerves [17], and changes to the soft tissue and associated structures [18]. Notably, reductions in sensory function may occur, such as reduced or altered taste and smell experience, or reductions to mechanical function, such as mechanical mastication and overall facial and neck movement [17,18]. To reduce the direct risk of malnutrition following surgery, the implementation of Enhanced Recovery After Surgery (ERAS) protocols should be considered, as they have been demonstrated to increase the time to solid food consumption following surgical procedures [19].

### 3.2. Radiotherapy

One of the primary modes of treatment used (approximately 75% utilization) in the HNC population is RT [13], and is often used in adjunct with other treatment forms [20]. Administration of RT is site-specific and localized, causing direct damage to all cells (including healthy cells) in this area. Consequently, damage to the structures involved with the consumption of food and for the early stages of digestion (i.e., salivary glands) are also inadvertently affected. This reduces the capability of adequate early digestion processes, as well as changes in taste perception of different foods. In combination with other side effects such as gastrointestinal upsets and loss of appetite, the effects of RT can also result in the inhibition of patients with HNC’s ability and desire to consume food. Fortunately, the application of intensity-modulated radiotherapy (IMRT) has been demonstrated to reduce RT-related toxicities [21], with this treatment being increasingly optimized [22].

### 3.3. Chemotherapy

Commonly utilized in combination with RT is CT, in particular cisplatin [23]. This treatment is often utilized as adjunctive therapy to ensure that all neoplastic cells are removed following surgery [24], or combined with RT in the case of metastasized cancer as an efficient method of treatment distribution to all tumor cells [24]. Use of CT can also lead to gastrointestinal upsets and loss of appetite, further resulting in the loss of desire to consume food, and can potentially lead to reduced efficiency in nutrient absorption [24]. However, the use of CT can exacerbate the toxicities caused by RT, such as oral mucositis, when used in combination [25].

## 4. Treatment-Related Toxicities

### 4.1. Oral Health Problems

The location of the initial tumor can have an impact on patients with HNC’s ability to consume food. Furthermore, up to 90% of patients experience symptoms that impact on their oral intake either due to the tumor location, or their prescribed treatments [26]. The treatments administered for patients with HNC are applied near many vital structural and functional organs responsible for the consumption of food. A relatively recent systematic literature review [27] has identified that these treatments cause multiple, interconnected side effects impacting on oral intake, further contributing to malnutrition. In addition, the participants reported changes in taste, and a reduction in desire to consume food due to appetite changes and pain sensations. Several side effects were also identified such as the development of oral mucositis (OM), xerostomia, dysphagia and dysgeusia.

#### 4.1.2. Oral Mucositis

Current oncology treatments are based on destroying the rapidly dividing cancer cells; however, this is also a characteristic of the oral cavity epithelium cells which are consequently disrupted or destroyed. Therefore, these treatments often result in the development of OM, negatively affecting the oral cavity and upper digestive tract [28]. The OM develops progressively, with early pathogenesis including an increased inflammatory response signaling the initiation of apoptosis. Consequently, this further leads to inflammation, resulting in erythema and edema of affected areas. As the condition develops, ulceration can also occur due to an increased risk of secondary infections at the site [25].

Although some patients with HNC may only experience the early stages of OM, approximately 60% of patients progress to the ulcerative stage, experiencing severe side effects, such as painful sites and an increased risk of infection [28]. In some severe cases, OM can lead to interruptions in active treatment, to allow for healing, impacting patient prognosis [29]. Severe OM is also associated with greater weight loss, reduction of energy and nutritional status [25], and while discomfort is experienced in all OM stages, the ulceration stage is most painful [30]. Additionally, OM development has the potential to begin immediately following the initial administration of cancer treatments [29], with patients with HNC receiving RT at a definitive risk of developing symptoms [31].

The optimal interventions for OM are orientated towards reducing the pain sensation, eradicating any pathogenic microbiota colonized in the area, and commencing the wound healing process, particularly at the site of any ulcerations (Table 1). Currently, there is no established ‘gold-standard’, widespread treatment for OM [32], however the most common and simplistic intervention utilized is the maintenance of oral hygiene [31], which should be adhered to throughout oncology treatment [33]. While there is limited evidence favoring any one oral health product [32], there is encouraging evidence for the use of sterile solutions, including saline, for oral hygiene maintenance [33]. Further interventions also include the use of honey, which has been successful in reducing the severity and duration of OM symptoms [34].

#### 4.1.3. Xerostomia

Xerostomia is a term used to describe dryness of the mouth, with approximately 80% of patients with HNC developing the symptoms [45]. Patients can develop xerostomia due to a surgical incision of their salivary glands, leading to an alteration in salivary function. However, the most common cause of this toxicity is the use of RT [45] due to the direct impact and damage to the salivary glands made by the active treatment [46], leading to a reduction in salivary production as well as cracking of the lips, tongue, and bleeding gums [47]. Xerostomia can also impact the patient’s social functioning due to changes in speech and causing psychological distress [46]. The functional changes causing reduced salivary levels can also contribute to a reduction in dietary intake [48].

While there is no consistent evidence available to support the use of any single treatment for xerostomia, various strategies are suggested for the management of its symptoms (Table 1). There are a variety of pharmacological systemic and topical agents available for patients who experience xerostomia, and these include a range of interventions that stimulate the salivary glands to increase saliva production (sialagogues) [37]. Similarly, some patients with HNC are prescribed the use of cytoprotective agents in concurrence with their RT or CT for the management of xerostomia symptoms [38]. However, patients have reported that the most beneficial relief solution for their xerostomia is frequent hydration [49].

#### 4.1.4. Dysphagia

The treatments for HNC can also have negative impacts on swallowing processes, due to the proximity of the treatments to the required physiological structures (i.e., tongue and cranial nerves) resulting in dysphagia [50]. Dysphagia can also lead to the functional tissue becoming fibrotic, which physically inhibits the swallowing process. In addition, surgical interventions can also lead to dysphagia, with its severity being dependent on multiple risk factors, including the primary tumor size and location, degree of surgical excision, and the requirement of surgical reconstruction [50]. The presence of dysphagia is directly linked to altered usual dietary intake [51]. Consequently, weight loss is identified as an independent predictor commonly associated with the presence of malnutrition in this population [48].

The management of the symptoms of dysphagia are focused on physical rehabilitation rather than the use of medicinal interventions (Table 1). In particular, swallowing therapies are successful in improving swallow function, leading to an improvement in oral intake [39]. The use of specific tongue strengthening exercises have also been found to reduce symptoms in HNC survivors, however further investigations are needed into the efficacy of these exercises in patients currently receiving treatment [40]. Patients also report the requirement for texture-modified foods, in particular the softening of foods through the addition of sauces, to allow for the consumption of an adequate diet [52]. In addition, it is speculated that the improvement of OM symptoms could improve the pathogenesis of dysphagia, such as through the reduction of fibrotic tissue [50].

#### 4.1.5. Dysgeusia

The administration of treatment can affect the taste receptors, with taste reportedly one of the main characteristics to be susceptible to alteration [53]. For patients with HNC receiving RT, there is no correlation observed between the dose of administered RT and taste sensation, with these changes also experienced prior to treatment due to loss of taste buds caused by surgery, or the tumor location [54]. Several changes in taste perception are reported, including the absence or incorrect perception of flavor; most commonly the perception of metallic, salty, and bitter tastes. The loss of taste associated with dysgeusia is also paired with a reduction in appetite and the enjoyment of food consumption [53]. Patients have also reported altering their usual food consumption to increase food palatability, including reducing consumption of foods with adverse flavors [51]. This can lead to an overall reduction in food intake, leading to a higher risk of malnutrition [55].

### 4.2. Gastrointestinal Upsets

Exposure to oncological treatments can result in a variety of gastrointestinal complications. These toxicities are more likely to be experienced by patients who are receiving CT, either in isolation or as CRT, in comparison to isolated RT [56], which adversely affects the tissue in the head and neck region, rather than the lower digestive tract. The symptoms of nausea and vomiting are reported simultaneously, potentially becoming so severe that patients may request the temporary discontinuation of their active treatments [57]. The rates of these side effects (nausea (75%) and vomiting (25%)) are considerably different in patients with HNC following CRT or CT, with nausea without vomiting commonly observed [56]. Further symptoms of gastrointestinal upset include diarrhea (3.5%) and constipation (10%), which are more likely to be caused by CT than RT [58]. However, the experience of constipation is more likely to occur due to the administration of anti-nausea medication than CT itself [59]. The altered lifestyle experienced during HNC treatment may also contribute to the development of constipation, through increasing a sedentary lifestyle, dehydration, and an altered dietary intake with a reduction in dietary fiber due to the changes occurring to patient’s oral health [60].

### 4.3. Pain and Fatigue

During HNC treatment, patients experience pain as one of the most commonly reported symptoms (up to 90%) experienced from the time of diagnosis [48] due to a variety of different reasons [61]. These can include; the effects of either the primary tumor or its subsequent metastases, pain from excision surgery, or as the consequence of the toxicities induced by RT and CT treatments, with the potential for pain to be experienced throughout the duration of the patients treatment [62]. Furthermore, this can result in a variety of negative health consequences, including problems related to nutritional intake, such as the ability to consume food and drink [50], with an increase in pain being inversely related with the low amount of energy consumed per day [48]. However, the reduction of the pain caused by treatment toxicities, such as reducing the severity of mucositis through the discussed measures, would assist in reducing the overall pain experienced in this population [61].

Fatigue is a commonly experienced side effect of oncology treatments, with emerging evidence suggesting that it is prevalent in nearly all patients with HNC [60]. While fatigue can be a direct effect of treatment, it has also been identified in patients prior to treatment commencement [63], and is associated with depression symptoms [64]. Fatigue has also been demonstrated to have an impact on the patients’ social functioning, including their motivation for social interactions and personal care, including the desire to source and prepare nutritional meals, and their ability to earn an income [65]. Despite the certainty of this population experiencing fatigue, it has been demonstrated that exercise, particularly resistance exercise, can reduce the experience of fatigue in this population [41,42].

### 4.4. Metabolic Alterations

The effects of the tumor and the treatments received by patients with HNC can also cause specific altered metabolic responses, including an altered resting energy expenditure [66]. These oncology treatments cause systemic inflammation, and play a role in the disruption of the metabolism of macronutrients, consequently causing changes in energy expenditure requirements [67]. The alterations in metabolism, in combination with reduced food intake causing a negative energy and protein balance, can lead to the development of cancer cachexia [68]. This is characterized by an ongoing loss of skeletal muscle, with or without the loss of fat mass, leading to progressive functional impairment that cannot be improved by conventional nutritional support [68,69]. A relatively recent exploratory study found a high prevalence of cachexia (42%) in patients with newly diagnosed HNC, with an additional 15% described as pre-cachexic [70]. Further, the presence of sarcopenia, or muscle wasting, is associated with the development of malnutrition, as well as the progression of treatment-related toxicities [71]. Sarcopenic obesity is also associated with poorer functional status and overall survival [72].

Patients with HNC who have an increased resting energy expenditure may not be able to achieve adequate nutritional intake due to other treatment side effects, such as oral health problems or gastrointestinal upsets. This may directly inhibit the consumption of the amount of dietary intake required, and contribute to the development of malnutrition [5], however there are nutritional interventions available to this population. The interventions most commonly utilized (Table 1) include oral nutritional supplements and nutritional counseling, which when used in combination have been demonstrated to result in better weight maintenance, increased protein and energy intake, improved QoL, and better tolerance to anti-cancer treatment [35]. In some cases, when patients with HNC are unable to consume an adequate dietary intake (60% of their estimated energy expenditure) for more than ten days, including due to an obstruction or severe toxicity such as OM, enteral feeding is also considered [36]. The use of parenteral nutrition is only utilized if the patient is unable to meet nutritional requirements orally or enterally [7]. The prevention or management of malnutrition through the minimization of treatment-related toxicities should be addressed from the initiation of active treatment, to prevent the requirement of these advanced dietary methods.

While nutrition interventions can assist patients in receiving the adequate nutrition they require, these interventions alone do not have an impact on the maintenance and development of lean body mass. The decrease in lean muscle mass that can occur during treatment can contribute to weight loss, and can be attributed to decreased physical function and QoL, and is associated with a treatment dose-limiting response, leading to extended periods of recovery [73,74]. The treatment-related toxicities experienced in this population are considered barriers to completing physical activity (PA) [75]. However, participation in PA programs, particularly resistance training, has been demonstrated to improve lean muscle mass (Table 1), including in late-stage and cachectic patients [43,44].

## 5. Consequences of Malnutrition

All of the oncology treatment side effects have the potential to contribute to the development of malnutrition in the HNC population [4]. It should be highlighted that the discussed treatment toxicities are not experienced in isolation, and they occur simultaneously with others. In addition, malnutrition in patients with HNC has the potential to lead to a cyclic exacerbation of the discussed treatment toxicities that play a role in its initial development [76]. Further, these toxicities, and the resulting malnutrition can cause further consequences, such as reduced QoL, that have the potential to negatively affect cancer prognosis.

### 5.1. Quality of Life

The treatment-related toxicities of xerostomia, dysphagia, gastrointestinal upsets, and fatigue have all been reported to impact QoL in patients receiving any oncology treatment [23], with OM identified to have a significant impact on QoL in patients within the HNC population [77]. The majority of patients with HNC experience a reduction in QoL, with the treatment side effects impacting on their normal physiological functioning, including their ability and motivation to consume food and drink [62]. Social functioning can also be impacted, including feelings of social isolation due to avoidance of social eating, fear of the requirement of tube feeding, and increasing financial difficulties and job loss [52,60].

### 5.2. Hospitalization and Treatment Interruptions

The dosage and intensity of the radiation administered, and the use of CRT are reported to be associated with an increased risk of hospitalization during treatment, with 60% of patients with HNC receiving CRT, and 45% of patients receiving isolated RT hospitalized [78]. Additionally, the oral health-related side effects can often be the reason patients are hospitalized or experience unplanned emergency department visits during active treatment [79]. Specifically, some of the more painful side effects of HNC and its treatments, such as OM, can lead to patient hospitalization [62], with approximately 10% of patients with HNC receiving CRT hospitalized for severe OM [80]. Patients have also required emergency medical attention for gastrointestinal upsets and dehydration, and have required hospitalization specifically for malnutrition-related symptoms [78,80]. Additionally, while patients with a percutaneous (PEG) or nasogastric (NG) tube experience a lower mean weight loss during RT, the use of enteral nutrition is also associated with a higher frequency of hospital admissions, including nutrition-related complications [36].

The administration of oncology treatment, particularly CRT, is a relatively consistent treatment regime, which can result in an inability of healthy tissue to adequately recover [45]. As a result, approximately 10% of patients with HNC were reported to request an interruption or alteration in their treatments to recover from treatment-related side effects, including reductions in the prescribed dosage per treatment [81]. Nevertheless, the pain experienced during severe OM is one of the most commonly reported causes for patients with HNC requesting to have their treatments interrupted, with up to 20% of interruptions the result of this toxicity [28,45]. Furthermore, the presence of malnutrition can have cyclic consequences, and cause a decrease in treatment response [79]. Treatment interruption can prolong the treatment time, and depending on factors such as tumor stage and treatment intensity, may have an impact on patient survival outcomes [82].

### 5.3. Psychological Distress

Psychological distress in cancer refers to the interference of the ability to cope with the physical symptoms of cancer and its treatment, with this distress including feelings of vulnerability and sadness, including depression, anxiety, social isolation, and mood disorders [83]. Malnutrition in patients with HNC is linked with signs of psychological distress, and includes adjusting to physical changes that may impact body image, such as rapid weight loss, or the impact of changes to the social meaning of eating [84]. Compared to other cancer types, patients with HNC have the highest prevalence of psychological distress, with a associations found between nutritional status, dietary intake and psychological status [85,86]. However, additional factors also contribute to psychological distress in this population, in particular anxiety in one-third of the population, and depression, experienced by 15–50% of patients [87]. Factors that are associated with anxiety throughout treatment are further fueled by the sensation of pain and social losses, while weight loss and speech problems are more commonly associated with depression [87].

## 6. Future Directions and Limitations

This review draws together multiple domains related to both the significant treatment-related causes of malnutrition in HNC, and the consequences malnutrition and these toxicities may have. Further research should focus on the minimization of these toxicities through determining the efficacy of the discussed interventions, such as determining the optimal dosage of treatments for the oral health complications. Additionally, a focus should be placed on the early intervention of these side effects during HNC treatment, to reduce the severity of these toxicities, and minimize the risk of malnutrition. Research should also focus on ways to minimize the impact of the consequences of these malnutrition-related toxicities. For example, a focus should be made on minimizing the impacts of malnutrition-related psychological distress through the exploration of social support, and methods to increase overall QoL in this population. Additionally, it has been demonstrated that patient education regarding the treatments and treatment-related toxicities that may develop is effective in informing and preparing patients, particularly on general oral health care measures [88]. The most effective method of patient education for the treatment toxicities and available preventative interventions should be investigated to determine the effect in reducing these toxicities, and therefore the incidence of malnutrition.

The present research is a comprehensive narrative review, which is met with several limitations. Firstly, several components of the review were not discussed in extensive detail, with the purpose of the review being to provide a broad summation of a variety of treatment-related toxicities and their involvement in the development of malnutrition. Secondly, while care was taken to include studies of a high quality and varied perspectives, selection bias exists through not completing a thorough systematic search of the literature.

## 7. Conclusions

Malnutrition is a multifaceted and debilitating condition which affects up to 90% of patients with HNC during active treatment. Due to the nature and location of these cancers, patients can experience a range of treatment-related toxicities which are directly attributed to the development of malnutrition. Toxicities include gastrointestinal upsets, pain and fatigue, as well as metabolic changes impacting overall health, leading to a decreased consumption of food. Oral health issues and treatment toxicities significantly affect the inability to consume food, directly increasing the incidence of developing malnutrition in people diagnosed with HNC. As identified in this review, malnutrition is extremely debilitating; it can negatively affect the severity of treatment-related toxicities, leading to reduced QoL, weight loss, and psychological distress, and have an impact on the patients’ ability to successfully complete active treatment. While there is considerable evidence for various interventions to minimize toxicity severity, particularly for oral health issues, it was identified that there is no consensus, and no gold-standard intervention for the treatment of these in patients with HNC. Therefore, further research should be completed to identify interventions that will allow for the minimization of treatment-related toxicities and patient reported symptoms, especially oral health issues. This would allow for an improvement in dietary intake, and reduce the incidence and severity of malnutrition in people diagnosed with HNC, improving their treatment experience, treatment and health outcomes, and quality of life.

## Figures and Tables

**Table 1:** The available interventions to improve treatment toxicities in patients with head and neck cancer (HNC) to prevent the development of malnutrition.

Side Effect	Intervention	Description	Objective of Intervention
**Altered dietary intake**	Nutritional therapies	Nutritional counseling [35]	Weight maintenance and increased protein and energy intake.
		Oral nutritional supplements (ONS) [35]	
		Enteral nutrition; Percutaneous or nasogastric tube feeding [36]	Administered in the presence of an obstruction or severe toxicity, or when inadequate dietary intake is consumed (60% of their estimated energy expenditure). Improves nutritional intake, however, is associated with hospital admissions.
		Parenteral nutrition [7]	Only required when HNC patient is unable to meet nutritional needs orally or enterally.
**Oral Mucositis**	Oral hygiene	Tooth brushing, flossing, and alcohol-free mouthwashes [31]	Ensures basic dental hygiene is met to reduce the severity of mucositis symptoms.
	Sterile solutions	Saline and sodium bicarbonate [33]	Maintenance of oral hygiene to compliment additional interventions to reduce mucositis symptoms.
	Traditional therapies	Honey [34]	Reduction in severity and duration of severe symptoms, weight maintenance and weight gain.
**Xerostomia**	Systemic and topical sialagogues	Medications that promote the secretion of saliva [37]	Improvements in salivary flow, decrease in requirement of oral comfort agents such as artificial saliva.
	Cytoprotective agents	Aim to protect healthy tissue from oncology treatments [38]	Reduction in 50% of clinical xerostomia cases.
**Dysphagia**	Physical rehabilitation	Swallowing therapies; motor exercises and swallowing maneuvers [39]	Improves swallow function and leads to improved oral intake.
		Tongue strengthening exercises [40]	Reduces symptoms; only evidence for HNC survivors.
**Reduced Exercise**	Resistance training	Increase in skeletal muscle and lean body mass [41,42,43,44]	Improvement in fatigue and acute QoL symptom management.
